# Clinical characteristics and prognosis of paediatric respiratory syncytial virus-related encephalopathy

**DOI:** 10.1186/s13052-024-01705-x

**Published:** 2024-07-29

**Authors:** Yushan He, Gang Liu, Xiuwei Zhuo, Xiaojuan Tian, Jun Liu, Xiaomeng Xu, Suyun Qian

**Affiliations:** 1 Pediatric Intensive Care Unit, Beijing Children′s Hospital, Capital Medical University, National Center for Children′s Health, Beijing, 100045 China; 2 Department of Neurology, Beijing Children’s Hospital, Capital Medical University, National Center for Children′s Health, Beijing, 100045 China

**Keywords:** Respiratory syncytial virus, Encephalopathy, Acute necrotizing encephalopathy, Acute brain swelling

## Abstract

**Background:**

To understand the clinical characteristics and prognosis of respiratory syncytial virus (RSV)-related encephalopathy in children.

**Methods:**

A retrospective analysis of the data of children who were diagnosed with RSV-related encephalopathy and admitted to the paediatric intensive care unit (PICU) of Beijing Children’s Hospital between November 2016 and November 2023 was performed.

**Results:**

Four hundred and sixty-four children with RSV infection were treated in the PICU, and eight of these patients (1.7%) were diagnosed with RSV-related encephalopathy. The mean age of the patients was 24.89 (5.92 ∼ 36.86) months. Two patients had underlying diseases. The time from the onset of illness to impaired consciousness was 3 (1.88–3.75) days. Five patients had convulsions, and three patients had an epileptic status. The serum procalcitonin (PCT) level was 1.63 (0.24, 39.85) ng/ml for the eight patients, and the cerebrospinal fluid (CSF) protein level was 232 (163 ∼ 848) g/L. Among the 8 patients, four patients underwent electroencephalogram (EEG) monitoring or examination. One patient showed continuous low-voltage, nonresponsive activity, and another patient displayed persistent slow waves, the remaining two patients had negative results. One patient had a combination of acute necrotizing encephalopathy (ANE) and acute encephalopathy with biphasic seizures and late reduced diffusion (AESD). Additionally, one patient had ANE, and another had acute brain swelling (ABS). One patient died in the hospital, and the other seven patients were discharged with improvement. Routine follow-up was conducted for 4.58(0.5 ∼ 6.50) years, and all patients fully recovered.

**Conclusions:**

RSV-related encephalopathy could have varying clinical manifestations, and some types, such as ANE and ABS, are dangerous and can lead to death.

## Background

Respiratory syncytial virus (RSV) is one of the most common pathogens of children [1, 2]. More than 95% of infants and children aged less than two years have a history of RSV infection [3]. Although most cases of RSV infection are mild, approximately 5.3% of children aged less than five years with RSV-related acute lower respiratory tract infection need to be hospitalized [4]. 8% of hospitalized children need to be transferred to the paediatric intensive care unit (PICU) [1], and the incidence of all-cause mortality in the PICU is approximately 2% [4]. The respiratory system is the most commonly damaged target organ in children infected with RSV, and extrapulmonary symptoms in severe cases of RSV infection have not been adequately studied. A previous systematic review [5] reported that 1.1–7.1% of children infected with RSV have neurological complications, and as many as 6.6–36.4% are treated in the PICU. Additionally, encephalopathy is relatively prevalent [6]. To date, there has been relatively limited research on RSV-related encephalopathy, with predominantly monocentric small sample studies [7–11]. Currently, research findings indicate that the severity of neurological complications caused by RSV infection may not be related to the severity of lung involvement [12]. RSV-related encephalopathy is primarily characterized by excitotoxic brain damage [7], which could also lead to severe types of brain diseases with a poor prognosis and a risk of death [5].

Thus, in this retrospective study, the clinical characteristics of patients with RSV-related encephalopathy admitted to the Beijing Children’s Hospital PICU over the past seven years at a single centre were reviewed with the aim of increasing the understanding of this disease.

## Methods

The data of children admitted to the PICU of Beijing Children’s Hospital who were diagnosed with RSV infection and met the diagnostic criteria for infection-related acute encephalopathy (AE) were retrospectively studied, and follow-up data were obtained and evaluated [13]. The Beijing Children’s Hospital Institutional Review Board approved this study ([2023]-E-123-R) with a waiver of the requirement to obtain informed consent.

### Inclusion and exclusion criteria

(1) Inclusion criteria: ① Twenty-nine-day to 18-year-old children who were admitted to the PICU of Beijing Children’s Hospital between November 2016 and November 2023; ② children who met the 2021 diagnostic criteria for AE [13], that is, there was a disturbance of consciousness during the acute onset of infection (Glasgow Coma Scale (GCS) score < 11 points and duration was ≥ 24 h); ③ RSV infection was confirmed by the presence of RSV positive nucleic acid or antigen in throat or nasopharyngeal swabs.

(2) Exclusion criteria: ① Well-defined meningitis or encephalitis caused by RSV infection, ②encephalopathy caused by other pathogens, and ③ a diagnosis of epilepsy or other causes of a disturbance of consciousness.

### Data collection and follow-up

(1) Data collection: Patient data, including age, sex, onset manifestations, and GCS score, were collected through the hospital electronic medical records system. Auxiliary examinations: routine blood tests, including tests of C-reactive protein (CRP), procalcitonin (PCT), coagulation parameters, and a biochemistry panel; cerebrospinal fluid (CSF)-related examination; and others. Simultaneously, electroencephalogram (EEG) and cranial imaging data, as well as treatment measures, were collected. Body temperature data were axillary temperature measurements.

(2) Encephalopathy was diagnosed and classified according to the 2021 Guidelines for the Diagnosis and Treatment of Acute Encephalopathy in Childhood [13]: ① AEs caused by a metabolic disorder: classic Reye’s syndrome and congenital metabolic disorders; ② encephalopathies caused by a cytokine storm: acute necrotizing encephalopathy (ANE) and hemorrhagic shock and encephalopathy syndrome (HSES) (if children met the criteria for ANE, they underwent further ANE Severity Score (ANE-SS) testing) [14], ③ encephalopathy with convulsive status epilepticus (possibly due to excitotoxic brain damage): AE with biphasic seizures and late reduced diffusion (AESD); and ④ miscellaneous syndromes: other/unclassified encephalopathy.

(3) Follow-up and prognosis: The GCS score of the patient at discharge from the PICU was recorded. Surviving patients are routinely followed up through outpatient or internet-based clinics based on the severity of their illness and the convenience of medical care. Patients with abnormal imaging findings at discharge underwent re-examinavtion, and the follow-up results were recorded. Following the initiation of this research, telephone follow-up with surviving children were conducted on August 2023, and Pediatric Overall Performance Category (POPC) scores were assessed during these calls [15].

### Statistical methods

Using descriptive analysis, the measurement data of the bias distribution are presented as M (range).

## Results

### Demographic information and clinical manifestations

**Fig. 1:**
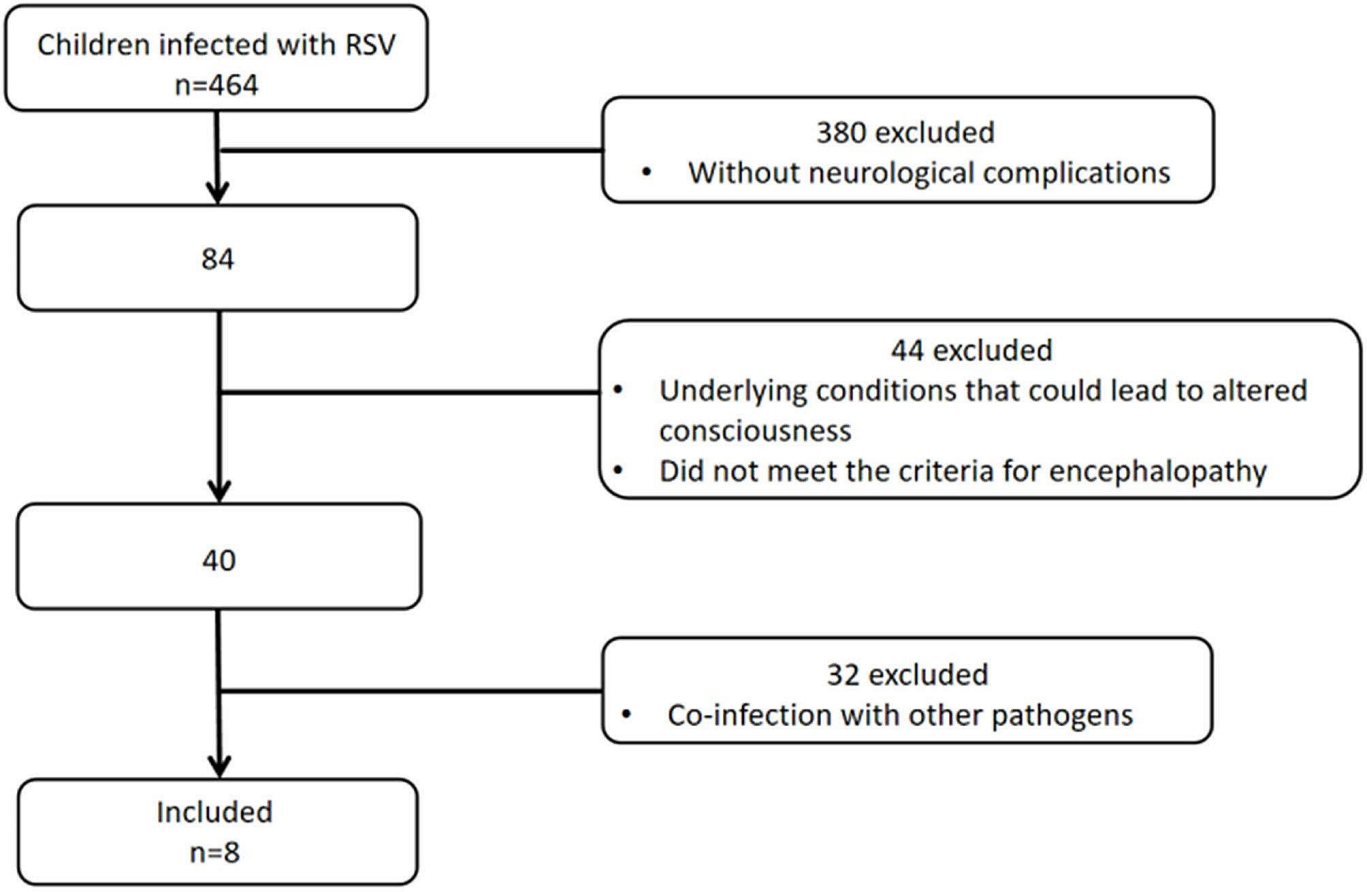
RSV-related encephalopathy inclusion and exclusion process

Four hundred and sixty-four children with RSV infection were admitted to the PICU of Beijing Children’s Hospital from November 2016 to November 2023. According to the inclusion and exclusion criteria, eight children (1.7%) with RSV encephalopathy were ultimately included (Fig. 1).

The children in this group were aged 24.89 (5.92 ∼ 36.86) months, and there were four boys and four girls. At the time of onset, six children had temperatures greater than 39℃, with a maximum temperature of 40.25℃ (39.03 ∼ 41.05℃). The time from the onset of illness to impaired consciousness was 3 (1.88 ∼ 3.75) days, and the GCS score was ≤ 8. Among the eight patients, five had generalized seizures, and six had complications (Table 1). Two children had an abnormal gestational history or underlying disease, Patient 7 had a 36-week premature delivery, and Patient 8 had a combined history of delayed intellectual and physical development and a ventricular septal defect. In the remaining children, no other abnormalities were found. The PICU hospitalization time was 7.5 (5 ∼ 11.5) days, and the total hospitalization time was 14 (10.5 ∼ 19) days.

**Table 1:** The clinical manifestations of patients with RSV-related encephalopathy

Characteristic	Patient 1	Patient 2	Patient 3	Patient 4	Patient 5	Patient 6	Patient 7	Patient 8
Age, m	27.53	0.97	1.87	27.27	22.50	39.97	41.93	18.07
Sex	Female	Male	Male	Male	Female	Female	Female	Male
Month of the year of onset	11	1	11	1	6	5	12	12
Onset symptom								
Maximum body temperature (℃)	40.0	38.1	36.0	40.5	41.2	41	38.5	39.2
Cough	+	+	+	+	+	+	+	+
Wheeze	+	+	-	-	-	+	+	+
Convulsion	-	-	+	-	+	+	+	+
Chemosis	+	-	-	+	-	-	-	-
GCS score at admission to the PICU	3	8	6T	5	5T	5T	7	7
Complications								
Central respiratory failure	+	-	+	-	-	-	-	-
Neurogenic shock	+	-	-	-	-	+	-	-
Hernia	+	-	-	-	-	-	-	-
Cardiac arrest	+	-	+	-	-	-	-	-
Status epilepticus	-	-	+	-	+	-	+	-
Multiorgan failure	+	-	-	-	-	+	-	-
Diagnosis	ABS	Unclassified AE	Unclassified AE	Unclassified AE	ANE superimposed on AESD	ANE	Unclassified AE	Unclassified AE

*ABS* acute brain swelling

### Laboratory findings

No obvious abnormalities in leukocytes were observed. Two patients had elevated CRP levels; five patients had elevated PCT levels, at 1.63 (0.24, 39.85) ng/ml; and four patients had significant increases in liver enzymes, with alanine aminotransferase (ALT) at 367 (10.8 ∼ 2385.78) IU/L. Three children had coagulation dysfunction, all of whom showed prothrombin time (PT) prolongation (Table 2). Patient 6 underwent venous cytokine and ferritin (Fer) examination, in which blood interleukin (IL)-1β, IL-6, IL-10, interferon-γ and Fer were elevated; blood IL-8, natural killer (NK) cell activity and soluble CD25 were normal; blood ammonia was 191 μmol/L; and blood sugar and lactic acid were normal. At the same time, her blood and urine metabolism screening tests indicated a decrease in guanidinoacetic acid. Whole-exome gene screening was performed for Patient 5 and Patient 6, and no abnormalities were found.

**Table 2:** Laboratory and imaging findings

Parameters	Patient 1	Patient 2	Patient 3	Patient 4	Patient 5	Patient 6	Patient 7	Patient 8
CRP (mg/dl)	1.40	3.00	<8	22.78	12.03	2.50	<8	<8
PCT (ng/ml)	70.71	0.47	1.21	2.78	35.36	41.34	0.16	0.48
PLT (10^9^/L)	41	280	272	187	118	37	273	247
ALT (U/L)	537.7	13.9	196.3	1.5	1671.6	4528.3	22.8	59.2
CK (U/L)	8101	54	387	50	222	917	82	182
CK-MB (U/L)	120	8	220	20	19	63	16	28
PT (s)	12.4	10.5	14.5	16.5	16.1	16.9	10.2	13.1
LP results								
CSF cells (×10^6^/L)	ND	0	0	2	2	5	6	0
Protein (mg/L)	ND	848	372	163	216	1068	116	232
EEG	Continuous low-voltage, nonresponsive activity	ND	ND	ND	Background front head or back head slow waves	Negative	Negative	ND
Brain CT	Widespread cerebral oedema, cerebral hernia formation	Right top blade-like low-density shadow, CT value 16Hu	Negative	Negative	Negative	Patchy area of low density was observed bilaterally in the thalamus	Negative	Negative
Brain MRI	ND	Negative	Negative	Negative	Bilateral cerebral hemisphere cortex swelling with subcortical leukocytic oedema and bilateral thalamic involvement	ND	Negative	Negative

*PLT* platelet count, *CK* creatine kinase, *CK-MB* MB isoenzyme of creatine kinase, *LP* lumbar puncture, *MRI* magnetic resonance imaging, *ND* not done

**Fig. 2:**
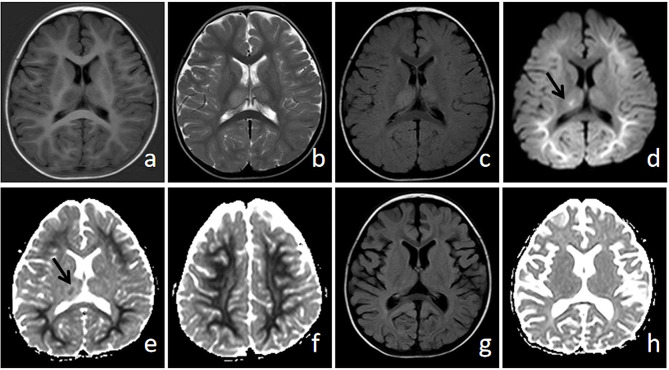
MRI results of Patient 5 One year and ten months old gairl, was admitted for “six days of cough, intermittent fever and convulsions for five days”. On the 8th day of the disease course, the brain MRI showed flaky long T1 (**A**) and long T2 (**B**) abnormal signals in the bilateral thalamus, T2 FLAIR (**C**) had a high signal, and small patchy diffusion restriction (**D**, **E** arrows) could be seen in the right thalamus lesion. Diffusion restriction (**D** ∼ **F**) could be observed in the bilateral cerebral hemisphere and subcortex, showing “bright tree signs”, indicating cytotoxicoedema. Re-examination of the brain by MRI on the 19th day of the disease showed brain atrophy-like changes in T2 FLAIR images (**G**), the abnormal signals in the bilateral thalamus had basically disappeared, and the apparent diffusion coefficient (ADC) (**H**) indicated that there was no restriction of diffusion in the brain

Nervous system-related examination: Patient 1 did not undergo lumbar puncture (LP) because of guardian refusal. The number of CSF cells in all the other seven patients was normal, and the CSF protein level was 232 (163 ∼ 848) g/L (Table 2). CSF smears and cultures were negative for seven patients, and CSF metagenomic next-generation sequencing (mNGS) test results for both Patient 5 and Patient 6 were negative. Moreover, five patients did not undergo CSF RSV-related testing. Four out of eight patients underwent comprehensive EEG monitoring or examninations. Patient 1 exhibited continuous low-voltage, nonresponsive activity on a bedside EEG. Patient 5 displayed slow waves activity on an EEG. Patient 6 and Patient 7 showed negative findings in routine EEGs on the third and ninth days after the onset of consciousness disorder, respectively. Brain MRI revealed “bright tree signs”, combined with bilateral thalamus involvement, in Patient 5. We considered that this patient had a combination of ANE and AESD (Fig. 2), with an ANE-SS score of 0 and a low risk. A diagnosis of ANE was considered for Patient 6, with an ANE-SS score of 7 (high risk).

### Treatment

Six children underwent tracheal intubation mechanical ventilation for 6.5 (4.75 ∼ 8.25) days, and two patients were treated with noninvasive ventilation for thirteen days and four days (Table 3). Six children were treated with glucocorticoid therapy at an initial dose of 2.5 (2 ∼ 8) mg/kg/d. Only Patient 6, who was diagnosed with ANE, received glucocorticoid therapy at an initial dose of 20 mg/kg/d and underwent plasma exchange therapy.

**Table 3:** Treatment and prognosis of patients with RSV-related encephalopathy

Findings	Patient 1	Patient 2	Patient 3	Patient 4	Patient 5	Patient 6	Patient 7	Patient 8
Glucocorticoids	-	-	+	+	+	+	+	+
IG	+	+	+	+	-	+	-	+
Antiviral drugs	+	+	-	+	-	-	+	+
Sedatives and anticonvulsive drugs	-	-	midazolam	midazolam	midazolam, clonazepam, levetiracetam, diazepam	midazolam, phenobarbital, levetiracetam, diazepam, propofol	phenobarbital, midazolam, diazepam	phenobarbital, propofol, diazepam, levetiracetam
Concentrated sodium chloride	+	-	+	+	-	+	-	-
Mannitol	+	-	+	+	+	+	+	+
Invasive ventilation time (d)	5	0	7	4	6	7	0	12
Vital status and GCS score at discharge	Death	15	15	15	14	14	15	15
Follow-up time (m)	-	6.50	4.67	4.58	0.50	0.50	6.50	4.50
POPC score at follow-up	-	1	1	1	1	1	1	1

*IG* immunoglobulin

### Follow-up and prognosis

Patient 1 was admitted to the hospital with a cerebral hernia after cardiopulmonary resuscitation due to central asphyxia on the 3rd day after onset. On the 7th day, her parents asked to stop treatment, and the patient died. The remaining seven children recovered and were followed up for 4.58 (0.50 ∼ 6.50) years. Brain MRI was conducted for children with imaging abnormalities in the last imaging examination during hospitalization, and the MRIs of Patient 5 and Patient 6 indicated brain atrophy.

## Discussion

Because of the limited number of reports of severe RSV-related encephalopathy, paediatricians often have a limited understanding of this disease. By retrospectively analysing the data of eight patients with RSV-related encephalopathy, we evaluated the clinical manifestations and prognoses in detail, thus providing a reference for paediatricians, especially paediatric intensive care physicians.

The incidence of RSV-related encephalopathy is relatively low; however, this condition can lead to AE with a high fatality rate, and the prognosis varies. Sweetman et al. [15] reported that the incidence of neurological complications in children hospitalized with RSV infection was 1.2%, and the incidence of neurological complications in children treated in the PICU was as high as 4.4–36.4% [16, 17]. Hon et al. [17] reported that among the neurological complications in children, 17.65% had encephalitis/encephalopathy, and no deaths were reported [16]. With a deeper understanding of the disease, reports of lethal encephalopathy caused by RSV infection have been increasing. Two cases of RSV-related ANE have been reported [10, 18], one of which involved serious neurological sequelae. HSES has also been reported [19]. In 2021, Saravanos et al. [5] systematically reviewed eight cases of death from RSV-associated neurologic complications, four of which seemed to be related to ABS, so vigilance was needed. In the present study, RSV-related encephalopathy was diagnosed in 1.7% of all children hospitalized with RSV infection in the PICU, including one patient with AESD superimposed on ANE, one with ANE, and one with ABS, among whom the one ABS patient died. Kawasaki et al. [8] reported that approximately 50% of children had residual sequelae or died, while the overall prognosis for surviving patients in this study was favourable. The prognosis of RSV-related encephalopathy reported by different investigators varies greatly, which may be related to the small sample size. Nevertheless, RSV-related encephalopathy can result in severe types of encephalopathy and poses a risk of death.

All patients in this study underwent CSF pathogen smear and culture, and no positive results were found. Two patients had negative CSF mNGS results. There are few reports of positive CSF pathogen test results in patients with RSV-related encephalopathy [7]. Morichi et al. [7] reported that among 9 patients with RSV-related encephalopathy, the CSF of 4 patients tested positive for RSV. It is speculated that the majority of patients may not have direct viral invasion of nervous system but rather an excessive immune response that causes damage.

The blood PCT level of children with RSV-related encephalopathy could increase to varying degrees and may serve as a reference for classification and prognosis. The PCT level could be used as a basis for determining whether children have an accompanying bacterial infection [20]. However, Maves et al. [21] noted that the PCT level is not sensitive for distinguishing between bacterial and viral infections in seriously ill children. Among our cohort, five patients had elevated PCT levels, with three patients showing notably high PCT levels, including those with ANE superimposed on AESD, ANE, and ABS. Moreover, the body temperature, ALT level, CK level, and levels of other indicators exhibited more pronounced increases in these 3 patients than in the other patients, accompanied by a decrease in the PLT levels. The patient with ABS died during hospitalization, while the other 2 children had relatively low GCS scores at discharge and recovered at 3 and 6 months after discharge. It could be speculated that these patients had a more pronounced inflammatory response contributing to a more critical condition.

The reasons for the elevated PCT in children with RSV-related encephalopathy remain unclear. The cause may be related to the elevation of cytokines such as IL-6 induced by viral infection, which stimulates high PCT expression [22]. Fujii et al. [23] found that blood PCT levels can also increase in patients with AESD, but no statistically significant difference in blood cytokine levels was observed compared to those in febrile seizure patients, suggesting that the elevated PCT levels in AESD patients may not be cytokine-induced. Nukui et al. [24] noted the presence of inflammatory responses in patients with ABS; however, further exploration is required to understand the reason behind the increased PCT levels. In conclusion, the initial degree of PCT elevation in children suffering from RSV-related encephalopathy may offer valuable insights into disease severity and prognosis. Nevertheless, additional research with a larger sample size is warranted for confirmation.

The types of RSV-related encephalopathy can be superimposed. Morichi et al. [7] reported that RSV-related encephalopathy was mainly caused by excitotoxic brain damage. According to the diagnostic criteria of the 2021 Guidelines for the Diagnosis and Treatment of Acute Encephalopathy in Childhood [13], the brain MRI of one patient in this study showed bilateral diffuse subcortical white matter cell toxic brain oedema with obvious “bright tree signs” and simultaneous involvement of the bilateral thalamus. The patient had ANE superimposed on AESD, that is, encephalopathy caused by excitotoxic brain damage and a cytokine storm. Cases of two types of encephalopathy induced by other pathogens occurring simultaneously in the same child have been reported [25], and this study is the first to report a similar phenomenon caused by RSV infection. This suggests that there might be multiple mechanisms involved in the occurrence of RSV-related encephalopathy, and further research is needed to elucidate its pathogenesis.

RSV infection can also induce ANE, and early positive treatment may improve patient prognosis. The mortality rate of ANE is approximately 30%; only 10% of children recover completely, and survivors often have severe neurological sequelae [6, 26–28]. To date, there have been no reported fatalities linked to RSV-related ANE. Yamamoto et al. [14] analysed the relationship between clinical symptoms and the prognosis of children and proposed that a high ANE-SS was strongly correlated with mortality and severe disability. In our study, a child with a low ANE-SS had a good prognosis after glucocorticoid treatment and six days of invasive ventilation. Another patient with a high ANE-SS was given invasive ventilation for seven days, and 20 mg/kg/d glucocorticoid, immunoglobulin and plasma exchange therapy were used on day 3 of the disease. At the follow-up, brain MRI indicated cerebral atrophy. The previous two reports of RSV-related ANE cases did not provide an ANE-SS, but the invasive ventilation time of these patients was significantly longer than that of the ANE patients in our study and that of one patient with serious neurological sequelae [10, 18]. The favourable outcomes in our study may be attributed to the timely administration of glucocorticoid therapy, as well as the potential impact of high-dose immunoglobulin and plasma exchange therapy [26, 29]. RSV-related encephalopathy can manifest as ABS, leading to death within a short period. Shiomi et al. [30] first proposed AE with ABS in 2003. The disease progressed rapidly, initially manifesting as symptoms related to cranial hypertension and signs of brainstem involvement within the following hours. Nukui et al. [24] reported the cases of four children with ABS, two of whom died. Lan et al. [31] reported that the incidence of outburst cerebral oedema in acute encephalitis was 2.4%, and the mortality rate reached 64%. All survivors were left with serious sequelae. Although these children were reported as having “acute encephalitis”, some of them may have had ABS. For most of the reported paediatric cases, ABS was induced by influenza virus infection. We found that RSV infection could also be a cause of ABS, and it may be relatively common among deceased children. Bottino [31] and Santos et al. [32] reported similar clinical characteristics in a child who died from RSV-related encephalopathy. In a systematic review, Saravanos [5] reported eight deaths from RSV-related encephalopathy, four of which were attributed to ABS [8, 11, 33, 34]. Xu et al. [11] reported a child, referred to as Patient 1 in this article, who had blurred consciousness on the third day of the disease, sudden central respiratory failure and coma, and cerebral hernia when he was admitted to the hospital. At present, the possible cause of ABS is rapidly progressive vasogenic oedema [24]. ABS is not completely consistent with the subtype of encephalopathy published in the 2021 edition of the AE Guidelines [13], and this type of encephalopathy has not been widely recognized. As it progresses rapidly, the prognosis is poor, and there is no clear effective treatment for this disease. Therefore, further attention and vigilance are warranted for this type of encephalopathy.

We classified encephalopathy and proposed, for the first time, the phenomenon of RSV-related encephalopathy overlapping with ANE and AESD. Currently, only 2 cases of RSV-related ANE have been reported, and this article presents the cases of an additional 2 patients. Furthermore, we confirmed that RSV can induce the occurrence of a fatal type of encephalopathy, especially ANE and ABS. Additionally, PCT may serve as an indicator of the type and severity of RSV-related encephalopathy.

However, there are some research limitations of this study. Firstly, this was a retrospective study with a relatively small sample size. Only 2 patients underwent CSF RSV testing, and some patients did not undergo MRI or EEG monitoring/examinations. In some patients, EEGs were negative findings indicative of encephalopathy, possibly due to the examinations being conducted during the recovery period of this disease and the short-duration of EEG examination, thus failing to capture the abnormalities that might have been present. In the future, multicentre prospective studies could be conducted to gain a more comprehensive understanding of RSV-related encephalopathy.

## Conclusions

This article summarizes the clinical characteristics of RSV-related encephalopathy and suggests that PCT could be utilized as an effective indicator for predicting the classification and prognosis of this condition. RSV-related encephalopathy can result in a poor prognosis for patients, and the occurrence of ANE and AESD in the same patient is a noteworthy phenomenon. Clinicians should be more vigilant about neurological complications that can occur following RSV infection, especially RSV-related encephalopathy. Furthermore, comprehending the underlying mechanism of RSV-related encephalopathy and the different types of encephalopathy is essential for improving treatment approaches and ultimately improving patient outcomes.

## Data Availability

The datasets used and/or analysed during the current study are available from the corresponding author on reasonable request.

## References

[1] Yu J, Liu C, Xiao Y, Xiang Z, Zhou H, Chen L, et al. Respiratory syncytial virus seasonality, Beijing, China, 2007–2015. Emerg Infect Dis. 2019;25(6):1127–35. 10.3201/eid2506.180532. PMID: 31107230; PMCID: PMC6537707. 31107230 10.3201/eid2506.180532 PMC6537707

[2] Hon KL, Leung AKC, Wong AHC, Dudi A, Leung KKY. Respiratory syncytial virus is the most common causative agent of viral bronchiolitis in young children: an updated review. Curr Pediatr Rev. 2023;19(2):139–149. 10.2174/1573396318666220810161945. PMID: 35950255. 10.2174/1573396318666220810161945 35950255

[3] Griffiths C, Drews SJ, Marchant DJ. Respiratory syncytial virus: infection, detection, and new options for prevention and treatment. Clin Microbiol Rev. 2017;30(1):277–319. 10.1128/CMR.00010-16. PMID: 27903593; PMCID: PMC5217795. 27903593 10.1128/CMR.00010-16 PMC5217795

[4] Li Y, Wang X, Blau DM, Caballero MT, Feikin DR, Gill CJ, et al. Global, regional, and national disease burden estimates of acute lower respiratory infections due to respiratory syncytial virus in children younger than 5 years in 2019: a systematic analysis. Lancet. 2022;399(10340):2047–64. 10.1016/S0140-6736(22)00478-0. Epub 2022 May 19. PMID: 35598608; PMCID: PMC7613574. 35598608 10.1016/S0140-6736(22)00478-0 PMC7613574

[5] Saravanos GL, King CL, Deng L, Dinsmore N, Ramos I, Takashima M, et al. Respiratory syncytial virus-associated neurologic complications in children: a systematic review and aggregated case series. J Pediatr. 2021;239:39–e499. 10.1016/j.jpeds.2021.06.045. Epub 2021 Jun 25. PMID: 34181989. 34181989 10.1016/j.jpeds.2021.06.045

[6] Hoshino A, Saitoh M, Oka A, Okumura A, Kubota M, Saito Y, et al. Epidemiology of acute encephalopathy in Japan, with emphasis on the association of viruses and syndromes. Brain Dev. 2012;34(5):337–43. Epub 2011 Sep 15. PMID: 21924570. 21924570 10.1016/j.braindev.2011.07.012

[7] Morichi S, Kawashima H, Ioi H, Yamanaka G, Kashiwagi Y, Hoshika A, et al. Classification of acute encephalopathy in respiratory syncytial virus infection. J Infect Chemother. 2011;17(6):776–81. 10.1007/s10156-011-0259-5. Epub 2011 Jun 7. PMID: 21647570. 21647570 10.1007/s10156-011-0259-5

[8] Kawasaki Y, Suyama K, Go H, Hosoya M. Clinical manifestations of respiratory syncytial virus-associated encephalopathy in Fukushima, Japan. Pediatr Int. 2019;61(8):802–6. 10.1111/ped.13928. Epub 2019 Aug 26. PMID: 31257673. 31257673 10.1111/ped.13928

[9] Morichi S, Morishita N, Ishida Y, Oana S, Yamanaka G, Kashiwagi Y, Kawashima H. Examination of neurological prognostic markers in patients with respiratory syncytial virus-associated encephalopathy. Int J Neurosci. 2017;127(1):44–50. Epub 2016 Feb 1. PMID: 26732732. 26732732 10.3109/00207454.2016.1138951

[10] Ong SCL, Nur Azidawati AH, Liew YH, Anita S. Acute necrotising encephalopathy of childhood: a review of two cases. Med J Malaysia. 2017;72(5):311–3. PMID: 29197889. 29197889

[11] Xu L, Gao H, Zeng J, Liu J, Lu C, Guan X, Qian S, Xie Z. A fatal case associated with respiratory syncytial virus infection in a young child. BMC Infect Dis. 2018;18(1):217. 10.1186/s12879-018-3123-8. PMID: 29751747; PMCID: PMC5948794. 29751747 10.1186/s12879-018-3123-8 PMC5948794

[12] Ng YT, Cox C, Atkins J, Butler IJ. Encephalopathy associated with respiratory syncytial virus bronchiolitis. J Child Neurol. 2001;16(2):105-8. 10.1177/088307380101600207. PMID: 11292214. 10.1177/088307380101600207 11292214

[13] Mizuguchi M, Ichiyama T, Imataka G, Okumura A, Goto T, Sakuma H, et al. Guidelines for the diagnosis and treatment of acute encephalopathy in childhood. Brain Dev. 2021;43(1):2–31. Epub 2020 Aug 20. PMID: 32829972. 32829972 10.1016/j.braindev.2020.08.001

[14] Yamamoto H, Okumura A, Natsume J, Kojima S, Mizuguchi M. A severity score for acute necrotizing encephalopathy. Brain Dev. 2015;37(3):322–7. 10.1016/j.braindev.2014.05.007. Epub 2014 Jun 12. PMID: 24931733. 24931733 10.1016/j.braindev.2014.05.007

[15] Fiser DH. Assessing the outcome of pediatric intensive care. J Pediatr. 1992;121(1):68–74. 10.1016/s0022-3476(05)82544-2. PMID: 1625096. 10.1016/s0022-3476(05)82544-2 1625096

[16] Kho N, Kerrigan JF, Tong T, Browne R, Knilans J. Respiratory syncytial virus infection and neurologic abnormalities: retrospective cohort study. J Child Neurol. 2004;19(11):859–64. 10.1177/08830738040190110301. PMID: 15658790. 10.1177/08830738040190110301 15658790

[17] Hon KL, Leung E, Tang J, Chow CM, Leung TF, Cheung KL et al. Premorbid factors and outcome associated with respiratory virus infections in a pediatric intensive care unit. Pediatr Pulmonol. 2008;43(3):275–80. 10.1002/ppul.20768. Erratum in: Pediatr Pulmonol. 2008;43(4):418-9. Hung, Erica [corrected to Leung, Erica]. PMID: 18219695; PMCID: PMC7168086. 10.1002/ppul.20768 PMC7168086 18219695

[18] Erdoğan S, Yakut K, Kalın S. Acute encephalitis and myocarditis associated with respiratory syncytial virus infections. Turk J Anaesthesiol Reanim. 2019;47(4):348–51. 10.5152/TJAR.2019.52028. Epub 2019 Mar 12. PMID: 31380518; PMCID: PMC6645850. 31380518 10.5152/TJAR.2019.52028 PMC6645850

[19] Uda K, Kitazawa K. Febrile status epilepticus due to respiratory syncytial virus infection. Pediatr Int. 2017;59(8):878–84. 10.1111/ped.13300. Epub 2017 Jun 28. PMID: 28423465. 28423465 10.1111/ped.13300

[20] Do Q, Dao TM, Nguyen TNT, Tran QA, Nguyen HT, Ngo TT. Procalcitonin identifies bacterial coinfections in Vietnamese children with severe respiratory syncytial virus pneumonia. Biomed Res Int. 2020;2020:7915158. 10.1155/2020/7915158. PMID: 32462018; PMCID: PMC7232683. 32462018 10.1155/2020/7915158 PMC7232683

[21] Maves RC, Enwezor CH. Uses of procalcitonin as a biomarker in critical care medicine. Infect Dis Clin North Am. 2022;36(4):897–909. 10.1016/j.idc.2022.07.004. PMID: 36328642. 10.1016/j.idc.2022.07.004 36328642

[22] Oberhoffer M, Stonans I, Russwurm S, Stonane E, Vogelsang H, Junker U et al. Procalcitonin expression in human peripheral blood mononuclear cells and its modulation by lipopolysaccharides and sepsis-related cytokines in vitro. J Lab Clin Med. 1999;134(1):49–55. 10.1016/s0022-2143(99)90053-7. PMID: 10402059. 10.1016/s0022-2143(99)90053-7 10402059

[23] Fujii Y, Yashiro M, Yamada M, Kikkawa T, Nosaka N, Saito Y et al. Serum procalcitonin levels in acute encephalopathy with biphasic seizures and late reduced diffusion. Dis Markers. 2018;2018:2380179. 10.1155/2018/2380179. Erratum in: Dis Markers. 2019;2019:4025694. PMID: 29725488; PMCID: PMC5872605. 10.1155/2018/2380179 PMC5872605 29725488

[24] Nukui M, Kawawaki H, Inoue T, Kuki I, Okazaki S, Amo K, et al. Clinical characteristics of acute encephalopathy with acute brain swelling: a peculiar type of acute encephalopathy. Brain Dev. 2018;40(9):792–8. Epub 2018 Jun 7. PMID: 29885875. 29885875 10.1016/j.braindev.2018.05.004

[25] Zhuo X, Ding C, Liu M, Dai L, Gong S, Tian X, et al. Clinical imaging features and prognosis of 40 children with influenza associated encephalopathy. Chin J Appl Clin Pediatr. 2021;36(24):1876–81. 10.3760/cma.j.cn101070-20210722-00866 10.3760/cma.j.cn101070-20210722-00866

[26] Bashiri FA, Al Johani S, Hamad MH, Kentab AY, Alwadei AH, Hundallah K, et al. Acute necrotizing encephalopathy of childhood: a multicenter experience in Saudi Arabia. Front Pediatr. 2020;8:526. 10.3389/fped.2020.00526. PMID: 33163461; PMCID: PMC7581867. 33163461 10.3389/fped.2020.00526 PMC7581867

[27] Okumura A, Mizuguchi M, Kidokoro H, Tanaka M, Abe S, Hosoya M, et al. Outcome of acute necrotizing encephalopathy in relation to treatment with corticosteroids and gammaglobulin. Brain Dev. 2009;31(3):221–7. 10.1016/j.braindev.2008.03.005. Epub 2008 May 5. PMID: 18456443. 18456443 10.1016/j.braindev.2008.03.005

[28] Song Y, Li S, Xiao W, Shen J, Ma W, Wang Q, et al. Influenza-associated encephalopathy and acute necrotizing encephalopathy in children: a retrospective single-center study. Med Sci Monit. 2021;27:e928374. 10.12659/MSM.928374. PMID: 33388740; PMCID: PMC7789050. 33388740 10.12659/MSM.928374 PMC7789050

[29] Li K, Zhang T, Liu G, Jin P, Wang Y, Wang L, et al. Plasma exchange therapy for acute necrotizing encephalopathy of childhood. Pediatr Investig. 2021;5(2):99–105. 10.1002/ped4.12280. PMID: 34179705; PMCID: PMC8212728. 34179705 10.1002/ped4.12280 PMC8212728

[30] Shiomi M. Influenzal encephalopathy. Nihon Rinsho. 2003;61(Suppl 2):100–6. Japanese. PMID: 12722196. 12722196

[31] Lan SY, Lin JJ, Hsia SH, Wang HS, Chiu CH, Lin KL, CHEESE Study Group. Analysis of fulminant cerebral edema in acute pediatric encephalitis. Pediatr Neonatol. 2016;57(5):402–7. Epub 2016 Jan 6. PMID: 26852357. 26852357 10.1016/j.pedneo.2015.11.002

[32] Santos PCP, Holloway AJ, Custer JW, Alves T, Simon L. Encephalitis and cytokine storm secondary to respiratory viruses in children: two case reports. Front Pediatr. 2023;10:1049724. 10.3389/fped.2022.1049724. PMID: 36741098; PMCID: PMC9895082. 36741098 10.3389/fped.2022.1049724 PMC9895082

[33] Kakimoto Y, Seto Y, Ochiai E, Satoh F, Osawa M. Cytokine elevation in sudden death with respiratory syncytial virus: a case report of 2 children. Pediatrics. 2016;138(6):e20161293. 10.1542/peds.2016-1293. Epub 2016 Nov 10. PMID: 27940684. 10.1542/peds.2016-1293 27940684

[34] Griffin N, Keeling JW, Tomlinson AH. Reye’s syndrome associated with respiratory syncytial virus infection. Arch Dis Child. 1979;54(1):74–6. 10.1136/adc.54.1.74. PMID: 420528; PMCID: PMC1545194. 420528 10.1136/adc.54.1.74 PMC1545194

